# Influence of the long-range ordering of gold-coated Si nanowires on SERS

**DOI:** 10.1038/s41598-018-29641-x

**Published:** 2018-07-27

**Authors:** Eleonora Cara, Luisa Mandrile, Federico Ferrarese Lupi, Andrea Mario Giovannozzi, Masoud Dialameh, Chiara Portesi, Katia Sparnacci, Natascia De Leo, Andrea Mario Rossi, Luca Boarino

**Affiliations:** 1 Nanoscience and Materials Division, Istituto Nazionale di Ricerca Metrologica, Strada delle Cacce 91, 10135 Torino, Italy; 2 Politecnico di Torino, Corso Duca degli Abruzzi 24, 10129 Torino, Italy; 3 Quality of Life Division, Istituto Nazionale di Ricerca Metrologica, Strada delle Cacce 91, 10135 Torino, Italy; 4 Instituut voor Kern-en Stralingsfysica, KU Leuven, Celestijnenlaan 200D, 3001 Leuven, Belgium; 5 Dipartimento di Scienze e Innovazione Tecnologica, Università del Piemonte Orientale Avogadro, INSTM, UdR Alessandria, Viale T. Michel 11, Alessandria, Italy

## Abstract

Controlling the location and the distribution of hot spots is a crucial aspect in the fabrication of surface-enhanced Raman spectroscopy (SERS) substrates for bio-analytical applications. The choice of a suitable method to tailor the dimensions and the position of plasmonic nanostructures becomes fundamental to provide SERS substrates with significant signal enhancement, homogeneity and reproducibility. In the present work, we studied the influence of the long-range ordering of different flexible gold-coated Si nanowires arrays on the SERS activity. The substrates are made by nanosphere lithography and metal-assisted chemical etching. The degree of order is quantitatively evaluated through the correlation length (*ξ*) as a function of the nanosphere spin-coating speed. Our findings showed a linear increase of the SERS signal for increasing values of *ξ*, coherently with a more ordered and dense distribution of hot spots on the surface. The substrate with the largest *ξ* of 1100 nm showed an enhancement factor of 2.6 · 10^3^ and remarkable homogeneity over square-millimetres area. The variability of the signal across the substrate was also investigated by means of a 2D chemical imaging approach and a standard methodology for its practical calculation is proposed for a coherent comparison among the data reported in literature.

## Introduction

Surface-enhanced Raman spectroscopy (SERS) is a powerful vibrational spectroscopic technique for chemical and biological sensing; it can be employed for molecular identification and trace analysis due to its high specificity and sensitivity^1,2^. The name itself emphasises the cornerstone of this technique: the amplification of Raman fingerprints of molecules interacting with noble metal nanostructures. While the enhancement is traditionally attributed to two factors, i.e. the electromagnetic and the chemical one, the first is widely accepted to give the largest contribution. Indeed, when a probe molecule is in close proximity to the surface, it profits from large local field enhancements that occur when localised surface plasmon resonances (LSPR) are excited^2,3^. In this surface-sensitive technique, the morphology of metallic structures plays a fundamental role in supporting the strong plasmonic enhancement. It determines the presence of localised regions called hot spots, found in between plasmonic objects with features size and spacing at the nanoscale, that are characterised by extremely large amplification of the electromagnetic near field^4^. The hot spots account for the largest enhancement of the Raman signal across the substrate and are responsible for a considerable part of the average enhancement factor (EF). There is a strong discrepancy between the enhancement of the signal from the molecules in these regions and in other locations characterised by a weaker near field intensity^5^. Substrates owning large EF often show poor uniformity and reproducibility of the measurement and inconsistent sensitivity, while substrates with significant uniformity show limited enhancement^2^. It is therefore clear that finding a fair compromise between EF and substrate uniformity is the key element for the realisation of an excellent SERS substrate.

Most of the research activity found in literature concerning the development of novel SERS substrates is mainly focused on the control of the location of hot spots with high density and uniform distribution. The purpose of these studies is indeed to guarantee favourable chemical detection level, towards single molecule detection^6,7^, along with low variability throughout the surface^8–10^. In the last few years, flexible gold-coated nanowires (NWs) have been successfully proposed as innovative SERS substrates. The flexibility has been highlighted as an important parameter because it allows the nanowires to lean against each other and trap the molecules in the hot spots at the tip-to-tip site generating a strong enhancement^11,12^. Besides conventional lithographic techniques, such as electron beam lithography or focused ion beam patterning^13–15^, several advanced techniques have been investigated in order to obtain gold-coated NWs including nanoimprint lithography^12^, self-assembly (SA) of metallic nanoparticles^16^, diblock copolymers^17^ and nanospheres lithography (NSL). Among them, one of the most promising techniques for the fabrication of high aspect-ratio NWs is given by the combination of NSL and the metal-assisted chemical etching (MACE)^18^.

NSL is based on the self-assembly (SA) process of spherical colloids leading to the formation of single- or double-layered crystals exhibiting hexagonally close-packed (HCP) symmetry. Thanks to this capability, NSL represents a promising manufacturing tool for the cheap fabrication of regular arrays of planar^19,20^ or three-dimensional nanostructures^21^, including metallic nanotriangles^22^, nanopillars^23,24^, nanomeshes^25^ or more complex configurations such as the Moiré crystals^26^. There are two main reasons that make the HCP symmetry obtained by NSL particularly suitable to meet the requirements of high EF and surface uniformity of SERS substrates. First of all, according to Kepler’s conjecture, the maximum density of nanospheres (NSs) with the same diameter is achievable in HCP configuration. As already reported^11^, the density of plasmonic structures strongly affects the SERS activity of the substrate since, by increasing it, an increment in the number of hot spots is ultimately produced. In addition, the periodicity of HCP-ordered nanostructures leads to a uniform distribution of hot spots over the sample.

Nevertheless, the maximum extent of the HCP configuration is limited by the appearance of defects during the SA process. Typically the defects consist of groups of NS with 5–7 close neighbours, whose presence causes the appearance of grains with different size and orientation. As direct consequence, the presence of defects produces a decrease in the number of nanostructures per unit area and, correspondingly, of hot spots. In order to preserve both homogeneity and density of the nanostructures, it is therefore mandatory to increase the average grain size of the NSs and to obtain a SA monolayer, avoiding the formation of multiple layers on the substrate. These two requirements are mainly influenced by the peculiar method for the deposition of the NSs. To fulfil this task, several deposition techniques have been explored so far, including floating^27^, micropropulsive injection^28^, spin coating^29^ or Roll-to-Roll Langmuir-Blodgett^30^. Among all these possible techniques, spin coating is one of the most convenient because of its applicability to wafer-scale processes and simple utilisation. Despite the number of spin coating protocols proposed to increase the monolayer coverage area and to reduce defects in the six-fold coordination of NSs^31,32^, not much attention has been given to the quantitative analysis of NSs effective long-range ordering. Moreover, the current understanding of the effect of the degree of order of the nanostructures on the performances of SERS substrates is still incomplete and not univocal.

In the present paper we describe a systematic study aimed at understanding the connection between the degree of order achieved in gold-coated nanowires fabricated by MACE and the intensity of the SERS signal. The method that we identified to quantify the long-range ordering is the calculation of the correlation length *ξ*^33^. This method takes into account both the presence of defects in the HCP configuration and the grain size. The variation of the long-range ordering is achieved by influencing the evaporation rate of the aqueous solution in which the NSs are dispersed, ultimately responsible for the SA mechanism^34^. To this goal, samples with different spin coating velocity have been prepared and the corresponding *ξ* have been measured. The most representative samples were then used to produce gold-coated NWs samples on which we performed SERS measurements over square millimetres areas. The knowledge deriving from this study will serve for the fabrication of substrates with controlled and uniformly distributed hot spots showing advantageous EF with low variability and wide range of applications.

## Results and Discussion

### Order maximisation

The SA mechanism of NSs during spin coating is quite complex. The process is influenced by several factors, including the dispersion properties of the colloidal solution (i.e. weight fraction, solvent volatility and viscosity)^29^ as well as the dynamic conditions during spin coating^32^. The dynamic conditions are given by the rotational speed (*ω*) and the acceleration (*α*) adopted during the spin coating process. The modification of these parameters has a direct impact on the evaporation rate of the solvent in which the NSs are dispersed, on the centrifugal forces and the surface tension among the nanoparticles^34^ and ultimately on the efficiency of the SA process.

**Figure 1:**
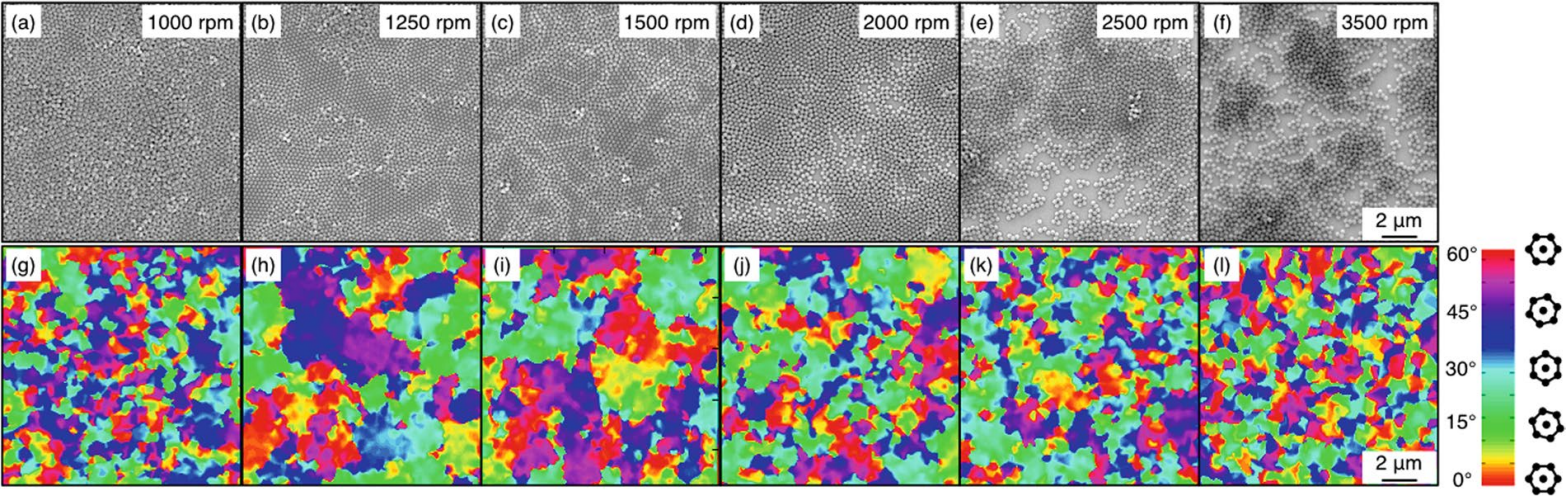
(**a**–**f**) SEM micrographs of the monolayer of polystyrene (PS) NSs on flat substrates achieved by spin coating at different speeds ranging from 1000 rpm to 3500 rpm. (**g**–**l**) The corresponding colour maps are shown under each SEM image.

In order to understand the relation between rotational speed and *ξ*, we prepared a set of samples in which we varied *ω* between 1000 rpm and 3500 rpm. We simultaneously varied *α* to maintain the ramp time constant to 3.05 s. For all the samples at different *ω*, we analysed the SEM micrographs (Fig. 1a–f), useful to evaluate the topographical distribution of NSs and to extract the values of *ξ*. By setting a spinning speed of 1000 rpm (Fig. 1a) we observed the formation of double layers of NSs, it is worth to notice that the HCP ordering of the second layer is often substituted by square close-packing, in this case it is not possible to perform any further lithographic process, thus such deposition parameter will be discarded in the subsequent experiments. By increasing *ω*, the layer of NSs becomes more compact and the formation of a monolayer is reproduced in the range between 1250 ≤ *ω* ≤ 2000 rpm (Fig. 1b–d). On the other hand, we observed the formation of an unpacked layer in all the samples processed at *ω* ≥ 2500 rpm, where large portions of the surface are not covered by the NSs, as reported in Fig. 1e,f. From a practical point of view, the speeds between 1250 rpm and 2000 rpm are adequate to perform a subsequent ordered pattern-transfer process.

**Figure 2:**
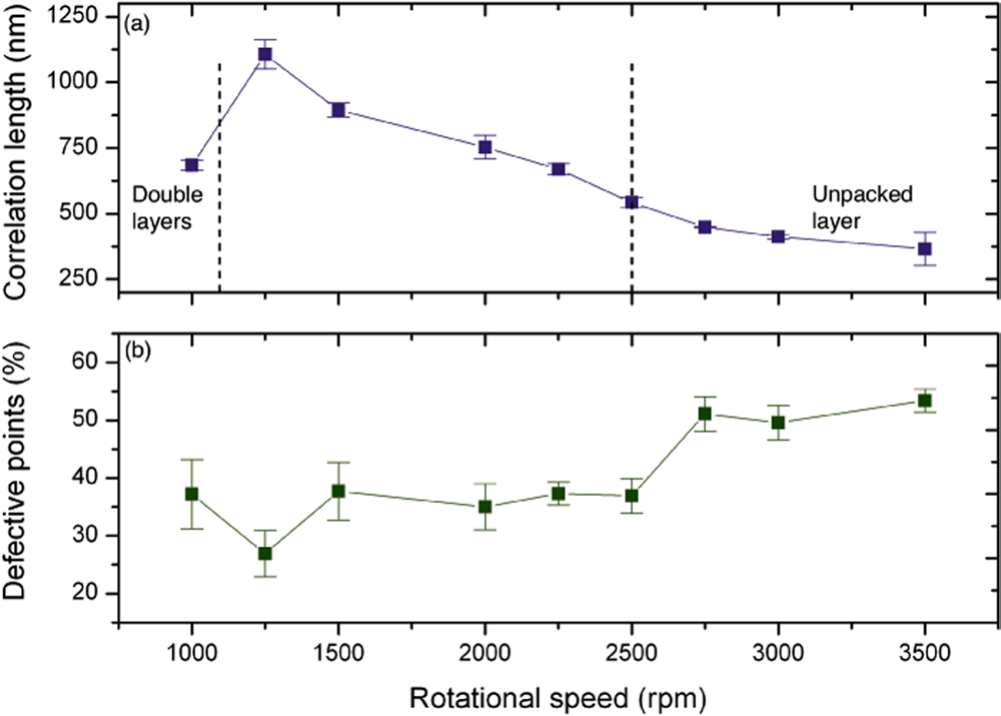
(**a**) The graph reports the monotonic decreasing trend of *ξ* at different rotational speed values. The maximum of *ξ* is found at 1250 rpm. (**b**) The graph shows the number of defects in the six-fold coordination of NSs in a monolayer on the substrate: the scatter plot refers to the sum of less and more the six nearest neighbours to each particle.

The quantitative analysis of the SA of NSs layers was achieved by image processing based on Delaunay triangulation of the SEM micrographs of the monolayer. We extracted the colour maps (Fig. 1g–l) representing the orientation of the grains and associate a *ξ* value to each spinning speed. This procedure highlighted the best deposition parameters to maximise the long-range ordering of the NSs. Figure 2a displays the behaviour of *ξ* calculated by the colour maps as a function of the rotational speed. For values in the range 1250 ≤ *ω* ≤ 3500 rpm, the obtained curve presents a monotonic decreasing trend with increasing spinning speed. For *ω* = 1250 rpm *ξ* reaches its maximum value 1084 nm and it drops to 339 nm for $\omega =3500$ rpm. The error bars are calculated as the standard deviation among *ξ* values extracted from different images for each substrate. The decrease of *ξ* to 1000 rpm can be associated to the presence of a second layer of NSs, as evident from Fig. 1a.

Every break in the periodicity of the matrix of NSs, due to the formation of domains, introduces a defective point in the triangulation. We counted the number of defects in the HCP configuration and we evaluated their total percentage. The graph in Fig. 2b marks that the percentage of defects in the monolayer follows a complementary trend as a function of *ω* with respect to *ξ*. For *ω* = 3500 rpm the number of defects reaches 55%; for smaller values of *ω* it decreases and presents a minimum value at the speed corresponding to the largest *ξ*. The value of *ξ* constitutes an indication of the ordering process and it can be considered representative of the whole sample. The effect of various dynamic conditions of spin coating on the ordering of NSs can be better understood through the comparison to a different SA system, such as the diblock copolymers (DBC). The ordering of DBC is influenced by the thermodynamic conditions during the thermal annealing process, i.e. time of annealing, temperature, heating and cooling ramp^35,36^. It has been observed that raising the temperature during the annealing process and tuning other parameters leads to a process of reorganisation of adjacent domains with the annihilation of defects^37^ to form larger and larger defect-free areas. Analogously, the effect of the rotational speed and acceleration during spin coating to which the NSs are subjected can be isolated by our analysis. By matching the graphs reported in Fig. 2, we are able to point out that a slow spin coating deposition contributes to trigger the evaporation of the solvent at a proper rate to activate the surface tension among NSs, resulting in the most correlated monolayer in our experimental conditions. On the contrary, at higher speeds the centrifugal force prevails on other physical interactions, spreading the nanoparticles on the substrate in random positions. The optimal dynamic conditions are dependent on the diameter of the NSs; we used nanoparticles with 250 nm in diameter, while the use of bigger NSs is often described in literature resulting in large domains^31^. Despite the significant interest for large area NSL and the use of *ξ* in DBC ordering estimate, the degree of order of SA NSs arrays have been evaluated only qualitatively rather than quantitatively through the correlation length, making it difficult to compare these results with previous works.

### SERS analysis on samples with different correlation length

**Figure 3:**
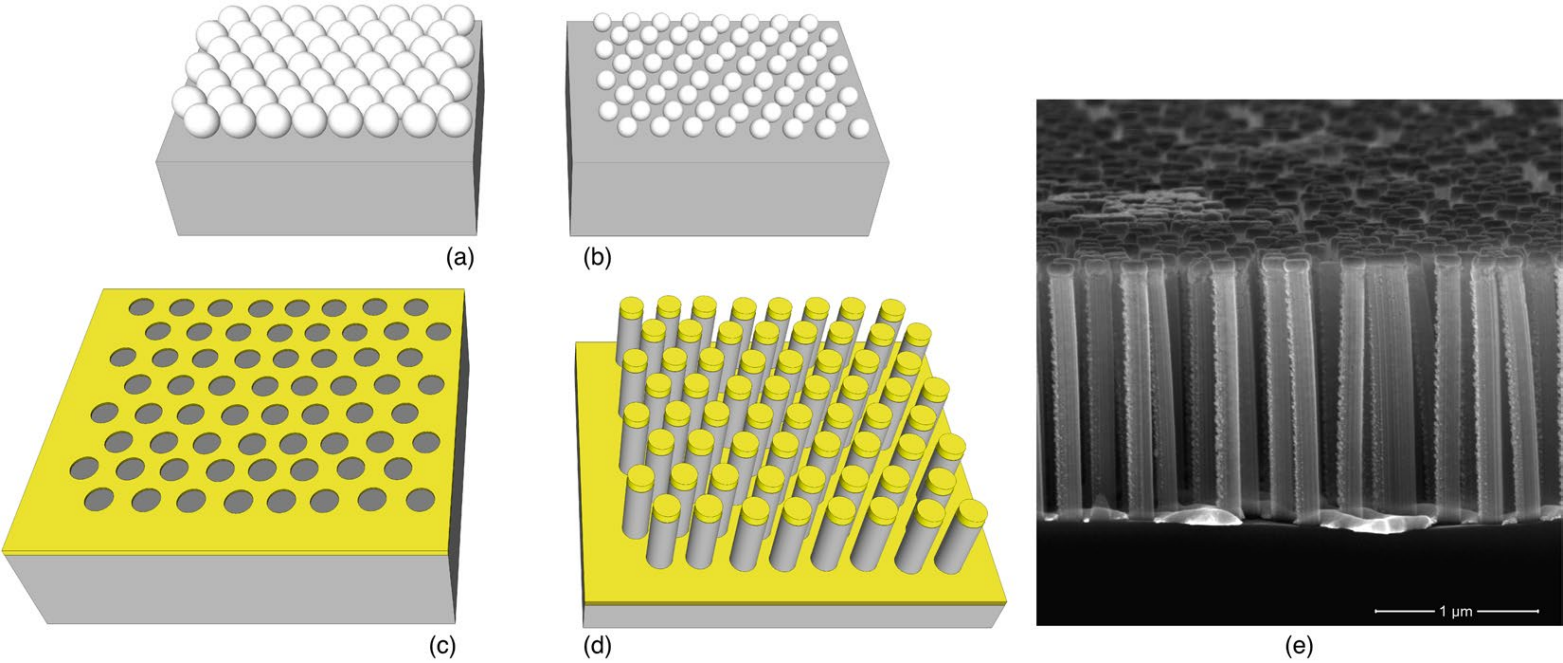
Scheme reporting each step of fabrication. (**a**) Deposition of PS NSs on the silicon wafer by spin coating at different rotational speed. (**b**) Reduction of NSs diameter by argon plasma. (**c**) Deposition of 20 nm of Au and lift-off of the NSs to obtain the pattern on the gold layer. (**d**) Fabrication of the nanowires by MACE and deposition of 100 nm. (**e**) SEM image showing the cross section of the definitive substrate prepared for SERS analysis, constituted of standing and flexible gold-coated silicon NWs.

Having determined the relation between the deposition parameters and *ξ*, we inspected the influence of degree of order on the activity of the SERS substrate. For the purpose of this test, we selected four samples with diverse values of *ξ* and in which the first step of deposition of the nanospheres was carried out by varying the spinning speeds. From this point on we will refer to these substrates as NW1 (*ξ* = 1084 nm and *ω* = 1250 rpm), NW2 (*ξ* = 861 nm and *ω* = 1500 rpm), NW3 (*ξ* = 757 nm and *ω* = 2000 rpm) and NW4 (*ξ* = 339 nm and *ω* = 3500 rpm). We completed the fabrication by means of NSs reduction by plasma etching and MACE to obtain a two-dimensional array of gold-coated NWs; Fig. 3 reports the fabrication steps and the SEM image of the resulting substrate. SEM images showing the substrates morphology after MACE are reported in Supplementary Fig. S1.

**Figure 4:**
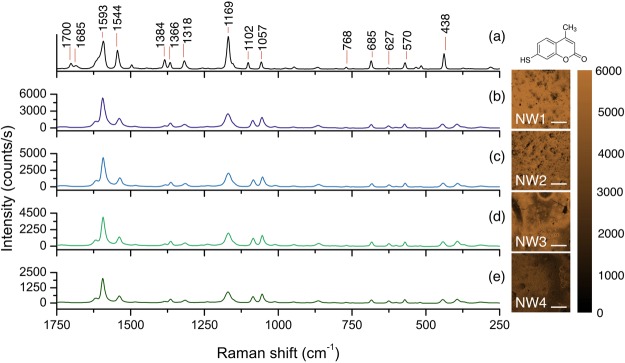
(**a**) Normal Raman spectrum of the probe molecule 7-mercapto-4-methylcoumarin (MMC). The Raman shifts are indicated above the main peaks in the spectrum and the corresponding assignments are reported in Table 1. The average SERS spectra of MMC acquired on the substrates (**b**) NW1, (**c**) NW2, (**d**) NW3 and (**e**) NW4 are shown. The chemical maps at 1593 cm^−1^ are reported for each tested substrate (the scale bars correspond to 0.5 mm).

For this experiment we used 7-mercapto-4-methylcoumarin (MMC) as probe molecule, because a strong covalent bond is formed between the sulfhydryl functional group (R-SH) and gold on the substrate. Normal Raman spectrum of MMC in solid state is shown in Fig. 4a. The major vibrational peaks, exhibited by the typical Raman fingerprint of pure MMC, are due to the stretching mode of the -C-O- bond at 1169 cm^−1^, to the conjugated -C=C- stretching mode at 1593 cm^−1^, and to the -C-C- stretching and in-plane deformation of -C-H_(*ring*)_ at 1543 cm^−1^. The band assignments to the other peaks, listed in Table 1, were obtained by a combination of a computational procedure with vibrational information from refs^38,39^.

**Table 1:** Band assignments to the Raman fingerprint of the MMC shown in Fig. 4a.

Raman shift (cm^−1^)	Assignment
438	Skeletal vibration
570	Ring vibration
685	Skeletal vibration
1057	Characteristic ring vibration
1102	Characteristic ring vibration
1169	-C-O- str
1318	-C-O- str.
1366	Conjugated -C=C- str. asym
1384	-C-H_3_ def.
1544	-C-C- str. and -C-H_(*ring*)_ in-plane deformation
1593	Conjugated -C=C- str. sym

After the incubation of the SERS substrates with MMC and setting the most efficient configuration, corresponding to leaning nanowires, micro-Raman mapping was performed on the four chosen samples within an area of 0.5 mm^2^ using a step size of 8 μm; the measurement was repeated eight times over each sample in a total area of 4 mm^2^. The spectra displayed in Fig. 4b–e represent the mean spectra of eight areas of analysis of 0.5 mm^2^. The resulting spectra show the features of MMC consistently with its conventional Raman spectrum. The major characteristic peaks cited above are clearly visible in their SERS spectral counterparts. However, some changes occurred in the relative intensities and Raman shift of some characteristic peaks of the MMC. In particular a significant decrease of the intensity was observed in the SERS spectra for the peaks at 438 cm^−1^, 1169 cm^−1^ and 1544 cm^−1^ shifted to 443 cm^−1^, 1171 cm^−1^ and 1539 cm^−1^, respectively. This is quite typical in SERS and it is mostly due to the interactions of the analyte with the surfaces of gold nanostructures, in particular to the orientation of analyte molecules on the substrate and their specific functional groups bound to the substrate^40^.

**Figure 5:**
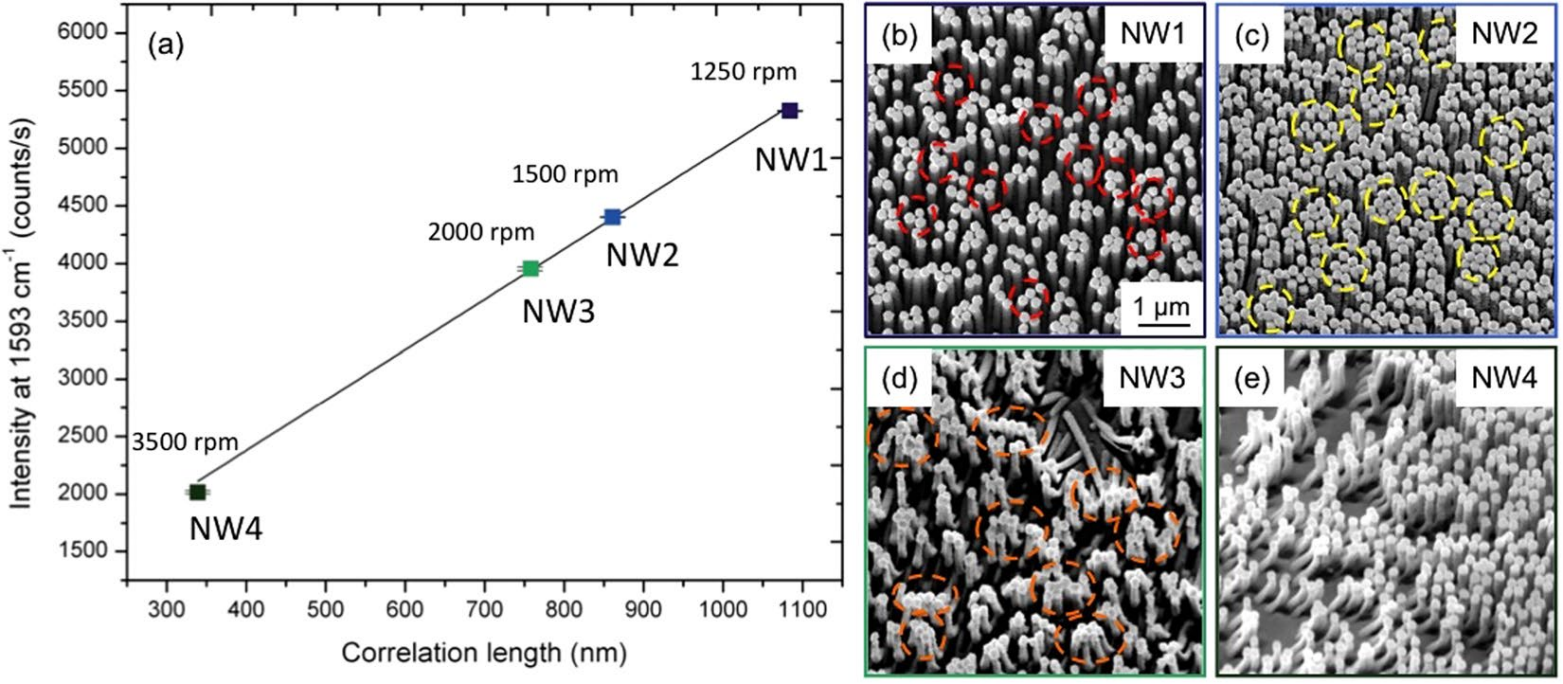
(**a**) Scatter plot of the average intensity of the SERS signal at 1593 cm^−1^ from the four characterised substrates as a function of the correlation length *ξ* of the nanostructures. (**b**–**e**) SEM micrographs representing the disposition of the nanowires after the probe molecule deposition.

The vibrational peak at 1593 cm^−1^ was used to compare the enhancement given by different substrates. We plotted the values of the intensities of the characteristic peak for the four substrates against the correlation length *ξ* of the original monolayer of NSs. As evident from Fig. 5a, the enhancing capability of our substrates is improved by the ordered nanowires; the intensity is enhanced up to 5300 counts/s in the substrate NW1 with the largest correlation length and it progressively falls for decreasing order reaching below half of this value for substrate NW4. The error bars report the relative standard deviation of the eight repeated measurements. The presented trend can be motivated by the increase in the NWs density and consequently of the hot spots in the most ordered substrate, coherently with the quality of the HCP configuration. However, the density alone is not enough to explain this result. The morphology of the hot spots has been investigated in the area with largest enhancement by matching the SERS maps to the SEM analysis. The images in Fig. 5b–e show the arrangement of the nanowires in the different substrates after the probe molecule deposition. In agreement with ref.^41^, the formation of bundles of two to five nanowires produces a greater enhancement of the electromagnetic field in proximity to the hot spot and this configuration is prevalent in the substrate NW1. The NWs bending configuration in substrates NW2 and NW3 is dominated by the formation of larger and irregular bundles leading to a lower enhancement of the SERS intensity. The bending configuration of the NWs is strongly conditioned by the degree of order on the substrate. A further evidence of the relevance of the degree of order *ξ* is given by the graph in Supplementary Fig. S2. The percentage reduction of the SERS signal, *ξ* and the NWs density are plotted for each sample and a strong agreement between the data sets for the SERS signal and *ξ* is evident. Thus the correlation length can be identified as a substantial and easily-measured parameter influencing both the density and the NWs bending arrangement playing a key role in the SERS signal enhancement.

The enhancement factor (EF) was calculated for the most ordered and efficient substrate NW1 according to the method proposed in refs^5,11,42^. Briefly, using the MMC mode at 1593 cm^−1^, we calculated the SERS EF of the gold-coated silicon NWs substrate at the excitation wavelength of 780 nm by applying the Eq. 1, where *I*_*SERS*_ and *I*_*Ref*_ are the intensities of the vibrational peak in SERS and normal Raman (NR) measurements, respectively, while *N*_*Ref*_ and *N*_*SERS*_ are the number of probe molecules in NR and SERS measurements.

1 $$EF=\frac{{I}_{SERS}{N}_{Ref}}{{I}_{Ref}{N}_{SERS}}$$

Since no Raman signal of the MMC was collected on a gold flat substrate (data not shown) due to the low sensitivity of the normal Raman in the detection of an organic monolayer, to establish the value for *I*_*Ref*_, a 0.01 M ethanol solution of MMC was measured. A detailed description on the method and the calculation of the EF is reported in the Supplementary Information for a more comprehensive clarification. An enhancement factor of 1.6 · 10^6^ was obtained. This value is in the same order of magnitude of the one reported in ref.^11^ on silver coated silicon nanopillars substrate.

The uniformity of SERS response over an active substrate is a crucial aspect in view of real application; in order to evaluate it we took into account the variability of the signal across the substrate through the relative standard deviation (RSD). The RSD is widely adopted in the SERS community to assess the spatial homogeneity of a substrate. Lower RSD values show remarkable homogeneity^43,44^ and values as low as 3% have been recently reported^45^ pushing the homogeneity to outstanding levels. Even though these figures are quite appealing for the application to analytical chemistry, it is worth considering that a detailed comparison is hindered by the fact that the practical calculation does not result from a standard protocol and every group reports its own methodology. Sometimes the RSD is calculated by considering the mean among tens to thousands punctual spectra^7,46^, while in other cases it is obtained from multiple scanning areas within a larger area on the substrate^10,47^. In some papers the scanning area onto which the analysis has been carried out is unclear^48,49^ leading to ambiguity in the results. For the purpose of defining the SERS substrates homogeneity for analytical and bioanalytical applications over large area, we suggest to extend the analysis to greater portions of the substrate and declare the analysed area unequivocally.

One interesting parameter to be considered is the intra-map homogeneity, which is well described by the RSD calculated on all the spectra composing the Raman map. In other words it represents the variability from pixel to pixel within one single map. As long as the scanned area is enlarged, the response variability of different sites increases, leading to a higher RSD value.

**Figure 6:**
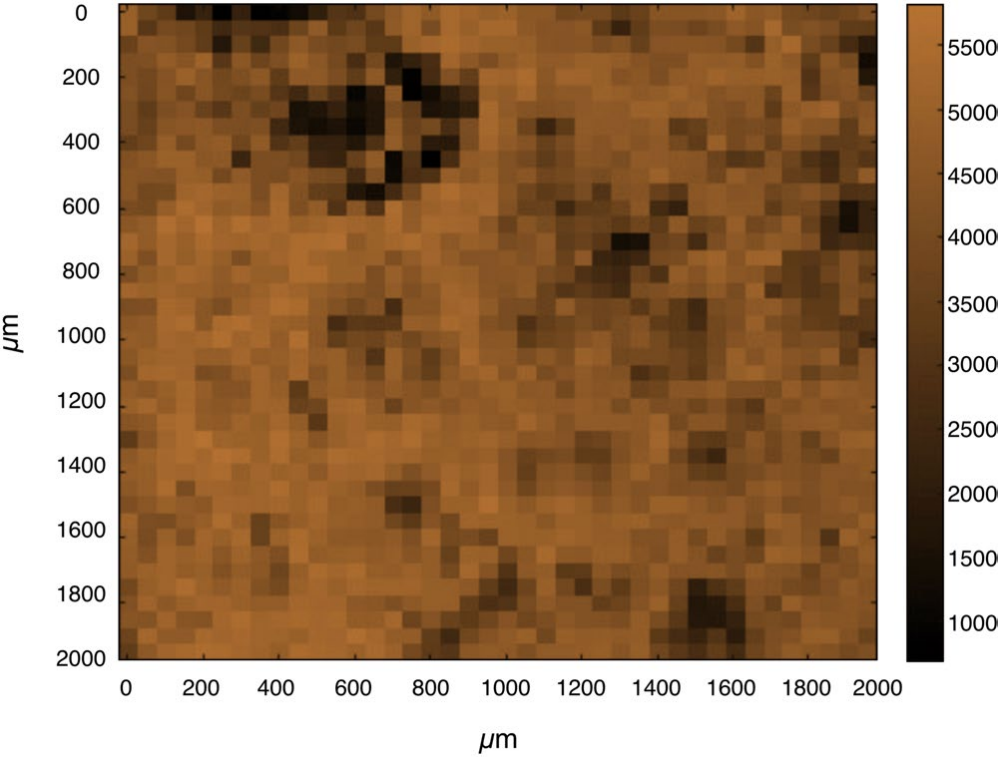
The chemical map at 1593 cm^−1^, acquired on the most ordered substrate NW1 with a step size of 50 μm on large area of 2000 × 2000 μm^2^, was used for the evaluation of surface homogeneity.

However, satisfactory repeatability of the measurements can be obtained by increasing the spot-size of analysis, in this way local differences are averaged. In punctual confocal Raman, the spot size is constant and it depends on the section of the focalised laser on the investigated surface (2.7 μm for 780 μm laser and 10x objective), however a spot-size enlargement can be practically obtained collecting spectra on a wider area and averaging all of them. In this way, the point-to-point intensity differences due to local defects can be overcome. The resulting mean spectrum is representative for the whole mapped area. In this concern, a relevant information is to define the minimum area that guarantees adequate repeatability. The RSD of repeated measurement of equal areas on the same SERS substrate, i.e. the inter-maps RSD, is considered to evaluate this. A similar approach is used by Novara *et al*.^50^ and Fu *et al*.^10^. For this scope, the calculation of the RSDs was performed by elaborating the SERS spectra acquired over progressively increasing areas from 150 × 150 μm^2^ to 2000 × 2000 μm^2^; the chemical map, shown in Fig. 6, is built over the maximum area of analysis around the band at 1593 cm^−1^. For each map dimension, four different regions of the substrate were scanned; the number of spectra included in the analysis is reported as well as the intra-map and inter-maps RSDs. The results and details of this analysis are specified in Table 2.

**Table 2:** Study of the substrate homogeneity through the relative standard deviation.

Scanned area (μm^2^)	Number of spectra	Intra-map RSD (%)	Inter-maps RSD (%)
150 × 150	9	12.1	12.8
300 × 300	36	16.4	13.6
450 × 450	81	16.2	10.7
600 × 600	144	17.4	8.0
750 × 750	225	18.1	7.3
900 × 900	324	17.9	6.8
1000 × 1000	400	18.1	6.6
2000 × 2000	1600	20	—

The intra-map RSD is the RSD calculated upon all the spectra composing one Raman map, the reported values are the mean of 4 determinations. The inter-maps RSD is the RSD calculated upon the four intensity averages for each map.

We found that the value of the intra-map RSD is 12.1% over the smallest scanning area and we registered a growth up to 20% for the maximum area of investigation. It is commonly accepted that RSD values around 20% indicate a fair grade of homogeneity in SERS detection^47,48,51^. On the other hand, the particularly interesting aspect is that the values of the inter-maps RSD decrease when the scanning area is enlarged. The inter-maps RSD remains around 13% for small areas, while it reaches values lower than 10% when the analysis is carried out over 500 × 500 μm^2^. Our result of about 13% for the intra-map and inter-maps RSD over 150 × 150 μm^2^ revealed an appreciable homogeneity level of the SERS substrate and the repeatability expressed by the inter-maps RSD values is a remarkable result. Moreover, our result constitutes an encouraging step forward in comparison to the homogeneity levels of 20% for substrates with leaning nanowires as declared by Lin *et al*.^45^ over a scanning area of 100 μm^2^.

In conclusion the application of SERS substrates to analytical chemistry requires the definition of a tailored and controlled fabrication protocol to ensure desirable enhancement together with homogeneity and reproducibility. The use of NSL and MACE for the fabrication of HCP-ordered matrix of flexible nanowires gave encouraging results. Matching the fabrication with a quantitative analysis offered an important understanding of the ordering process in NSL and the possibility to maximise *ξ* of the nanostructures up to 1100 nm. The increase of the correlation length led to growing enhancement, coherently with an ordered and dense distribution of hot spots, and to remarkable homogeneity over square-millimetres area, inter-maps RSD of 8% was calculated on 600 × 600 μm^2^. To broadening the field of applicability of this substrate, our future effort will be focused on upgrading the fabrication process to prevent the formation of grain boundaries and other irregularities that might weaken the homogeneity of the substrate over large areas. In particular, an additional topographical patterning will be developed on the silicon wafer to direct the self-assembly of NSs.

## Methods

### Materials and chemicals

Polystyrene NSs were synthesised by emulsion polymerisation (see ref.^18^ for details) with diameter equal to (250 ± 4) nm. Silicon wafers were purchased by SunEDISON/MEMC, silicon was doped with phosphorus, n+ type with resistivity 27–47 Ω cm and crystallographic orientation 〈100〉. Substrate functionalisation was obtained using piranha solution (H_2_SO_4_ (96%) and H_2_O_2_ (30%) with 3:1 ratio). The etching solution was made with HF (50%): H_2_O_2_ (35%): EtOH with 10:1:3 ratio in volumes. Ethanol, acetone and deionised water were used to rinse the samples. All reagents were purchased from Carlo Erba Reagents. SERS active molecule is 7-mercapto-4-methylcoumarin (C_10_H_8_O_2_S), purchased by Sigma-Aldrich.

### Nanospheres lithography

Sample preparation started by cleaning the silicon substrates (2.25 cm^2^) with acetone, ethanol and deionised (DI) water. Surface was made hydrophilic for the deposition of NSs aqueous solution by immersing the samples in piranha solution at 80 °C for 40 minutes and abundantly rinsing with DI water. We deposited the NSs using a programmable spin coater (Laurell Technologies) to modify the steps of the process. For each sample, we spread 60 μl of colloidal solution on the hydrophilic wafer. Our protocol consisted in two steps, the first lasts for 10 s where *ω* and *α* are set to 500 rpm (1 rpm = *π*/30 rad s^−1^) and 410 rpm/s, respectively. We varied the second step to find the best parameters as reported in the Results and Discussion. The resulting monolayer of NSs is treated with plasma etching (Bdiscom Plasma Matrix) using argon ions with 75 W RF power in a pressure of 1 Pa for 8 minutes. NSs are reduced in size and result in a non-close-packed monolayer that constitutes a shadow mask for the deposition of 20 nm of gold. Gold was evaporated by e-gun in a high-vacuum chamber (base pressure 5 · 10^−5^ Pa, distance from the crucible 10 cm, accelerating voltage 6 kV, filament current 35 mA and deposition rate 1.5 nm s^−1^). We lifted off the NSs in an ultrasonic bath of ethanol and obtained a pattern on gold with a two dimensional array of circular openings corresponding to the position of the spheres.

### Correlation length analysis

The quality of the NSs monolayer was investigated through the systematic acquisition of SEM micrographs for each sample by using a FEI Inspect-F field emission gun scanning electron microscope (FEG-SEM). Image processing and quantitative analysis was carried out by a MATLAB® tool. The software operates by identifying each NS within a given size interval and with a certain sensitivity level influenced by contrast and brightness of the image. Each particle is represented by a point in the HCP lattice and connected to all its nearest neighbours. Each point having less or more than six nearest neighbours, expected in the six-fold coordination, is identified as a defect^52^. The defects delimit the domains boundaries in the triangulation diagram, as shown in Supplementary Fig. S3. From this diagram, the software extrapolates a colour map wherein each colour variation corresponds to a different orientation of the domains. Elaborating the colour maps data generates the so-called auto correlation function *ACF* = *e*^−**r**/*ξ*^, where **r** is the vector indicating the location of the domains in the two-dimensional lattice^53^. The coherency of the order is preserved within a distance *ξ*, obtained through an exponential fitting of the ACF.

### Nanowires fabrication and SERS measurements

MACE was used for the fabrication of Si NWs^18,54^. Ethanol was used in the solution to increase the adhesion of the gold layer to the substrate^55^. We set the etching time to 210 s and then abundantly rinsed the sample with ethanol and let it dry in air. The result is a matrix of standing silicon NWs, with high aspect ratio of 1:10. This feature determines the flexibility of NWs. The ultimate substrate for SERS is obtained by coating the silicon NWs with 100 nm of gold on top as shown in Fig. 3e. Before depositing the probe molecule, we performed plasma cleaning in O_2_ (40 W, 1 minute) to remove any organic residue from the surface. The samples were immersed in a 1 mM solution of MMC in ethanol for 120 minutes and then rinsed with ethanol to remove any excess from the surface. They were subsequently rinsed with DI water to induce the bending of the NWs, due to capillarity effect arising during the evaporation of water because of its high surface tension coefficient, and to entrap the molecules in the hot spot regions^18^.

We carried out SERS measurement using a Thermo Scientific DXR™ xi Raman Imaging confocal microscope system equipped with an excitation laser source at 780 nm, a full range resolution grating of 5 cm^−1^ with a spectral range from 50 cm^−1^ to 3500 cm^−1^, a 10x microscope objective, a 50 μm pinhole aperture and an automatic x, y motorised stage. Each spectrum of the map was registered at 600 Hz (0.00167 s integration time) with 10 scans and with a laser power of 8 mW. The spectral maps were processed with MATLAB® for map unfolding, statistics and colour maps reconstruction based on the intensity of the peak of interest. The experiments for the enhancement factor calculation were carried out using the same instrumentation equipped with a 20x long working distance (LWD) microscope objective. Raman spectra were registered with an exposure time of 1 s for 20 scans in total and at a laser power of 8 mW. More details about the method and the calculation of the EF can be found in the Supplementary Information.

### Computational procedure

Geometry optimisation of model MMC structures and consequent calculations of vibrational (IR and Raman) spectra were carried out with DFT method using Gaussian 03 program^56^. Full geometry optimisations were carried out without symmetry constraints. Computations were performed with the Lee, Yang and Parr correlation functional (LYP)^57^ combined with the Becke’s non-local three-parameter hybrid exchange functional, (B3)^58^. Vibrational data were compared with the experimental Raman spectrum of MMC and the main bands in the spectrum were assigned^38^.

### Data availability

The datasets generated during and/or analysed during the current study are available from the corresponding author on reasonable request.

## Electronic supplementary material

Supplementary Information
