# Supplementary material for: Influence of the long-range ordering of gold-coated Si nanowires on SERS
Source: Sci Rep. 2018 Jul 27;8:11305. doi: 10.1038/s41598-018-29641-x (PMC6063917; doi:10.1038/s41598-018-29641-x)
Supplement: Supplementary file 1 — Supplementary Information [file 41598_2018_29641_MOESM1_ESM.pdf]

Supplementary Information
Influence of the long-range ordering of gold-coated Si nanowires on SERS.

Eleonora Cara[1,2,*], Luisa Mandrile[3], Federico Ferrarese Lupi[1], Andrea Mario Giovannozzi[3], Masoud Dialameh[1,2,4], Chiara Portesi[3], Katia Sparnacci[5], Natascia De Leo[1], Andrea Mario Rossi[3], and Luca Boarino[1]

[1]Nanoscience and Materials Division, Istituto Nazionale di Ricerca Metrologica, Strada delle Cacce 91, 10135 Torino, Italy
[2]Politecnico di Torino, Corso Duca degli Abruzzi 24, 10129 Torino, Italy
[3]Quality of Life Division, Istituto Nazionale di Ricerca Metrologica, Strada delle Cacce 91, 10135 Torino, Italy
[4]Instituut voor Kern-en Stralingsfysica, KU Leuven, Celestijnenlaan 200D, 3001 Leuven, Belgium
[5]Dipartimento di Scienze e Innovazione Tecnologica, Università del Piemonte Orientale Avogadro, INSTM, UdR Alessandria, Viale T. Michel 11, Alessandria, Italy
[*]e.cara@inrim.it

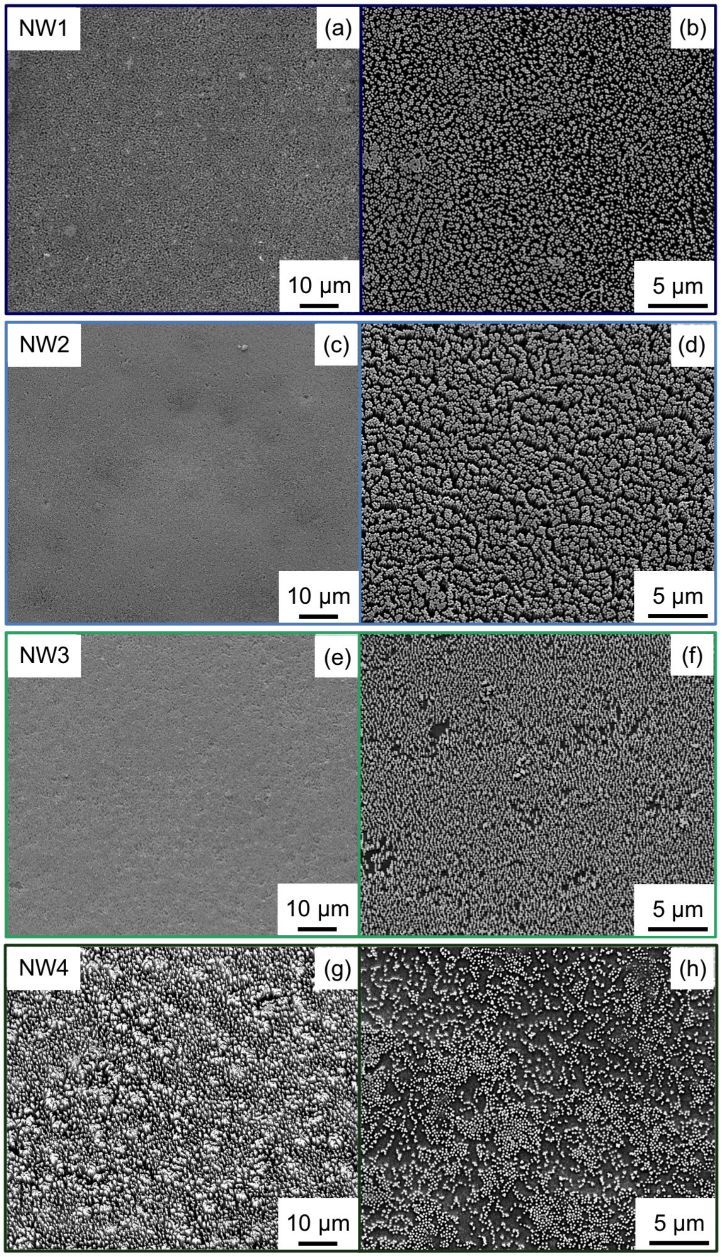

Figure S1. Two SEM images are reported for the four analysed samples (a)-(b) NW1, (c)-(d) NW2, (e)-(f) NW3 and (g)-(h) NW4 after the deposition of the probe molecule and the leaning of the nanowires. The SEM images show a top view of the sample at two different magnifications, thus providing a clear idea of the degree of uniformity of the substrates.

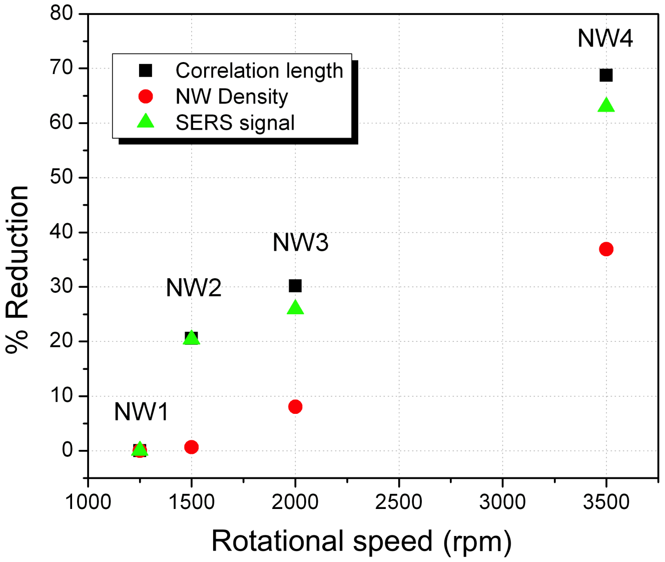

Figure S2. The scatter plot reports the percentage reduction of the correlation length, the nanowires density and the SERS signal for the four samples as a function of the rotational speed. Each data set was normalised to the measured value on the sample NW1. The number of nanowires per unit area is connected to the degree of order and increase for higher correlation length. Such quantity is taken into account as a parameter influencing the enhancement through the substrate. However, to clarify the relevance of the correlation length in the analysis, it is interesting to observe the strong agreement between the percentage reduction of the correlation length and that of the SERS signal intensities.

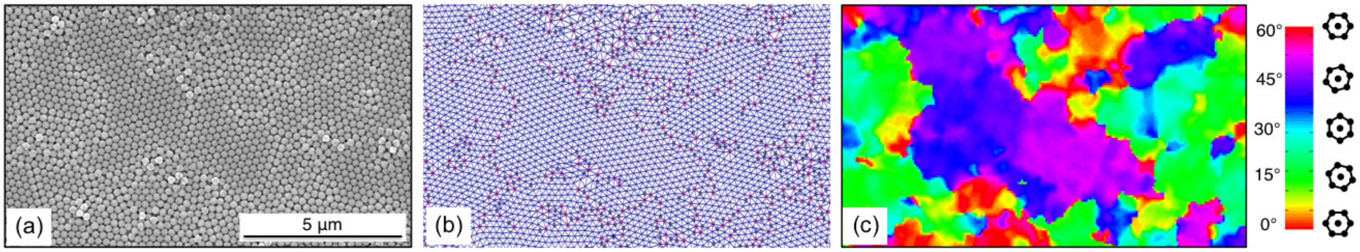

Figure S3. (a) The SEM image of the nanoparticles monolayer is shown. (b) The image processing tool based on Delaunay triangulation produces a diagram where each nanoparticle is identified as a point in the HCP lattice and it is connected to its nearest neighbours. The blue and red circles in the triangulation diagram specify the presence of defects where the nanoparticle is connected to five or seven nearest neighbours, respectively. (c) The different defect-free domains have random orientation highlighted by the use of a colour map.

Enhancement factor

The EF was calculated according to the method proposed in Ref 11, 42, 43 in the manuscript. Using the MMC mode at 1593 cm^{-1}, we calculated the SERS EF of the gold-coated silicon nanowires substrate at the excitation wavelength of 780 nm. We first determined the laser interaction/probe volume by placing a SERS substrate in the focus plane and then moving the substrate out of the focus plane in increments of 2 µm. The Raman intensity at 1593 cm^{-1} for each z-position was integrated and normalised by the maximum intensity at the focus plane, where the laser spot size was 1.9 µm in diameter. The probe volume is modeled as an ellipsoid with two axes equal to the laser-focus spot diameter and the height equal to the effective probe depth. Thus, the interaction volume using a 20X long working distance (LWD) objective (NA= 0.4) and a laser power of 8 mW was measured to be 67.8 μm^3. The Raman EF was calculated from the formula:

$$EF = \frac{I_{SERS} N_{Ref}}{I_{Ref} N_{SERS}}$$

The numbers used in the calculation were obtained using the methods described below. To establish the value for I_{Ref} a 0.01 M ethanol solution of MMC was measured with a 20X LWD objective, using an excitation wavelength of 780 nm and a power of 8 mW. 5.2 counts/s (I_{Ref}) were measured at the 1593 cm^{-1} peak. Using the probe volume of 67.8 μm^3, the number of molecules responsible for the Raman signal (NRef) was calculated to be $4.1 \cdot 10^8$ molecules. To establish the values for I_{SERS}, a 1x1 cm piece of gold coated silicon nanowires substrate (NW1), with a wires density of 14.8 μm^{-1}, was immersed into a 1 mM MMC solution in ethanol for 2 hours. Then the substrate was copiously rinsed with ethanol to remove the excess on unbound MMC molecules, rinsed with DI water to induce the leaning of the wires and let it dry in air. Using identical conditions as for I_{Ref} , a signal intensity of 65700 counts/s was measured for the 1593 cm^{-1} peak. The number of molecules inside the laser spot was estimated by assuming a monolayer of MMC on the tips of the gold-coated silicon NWs. An area of $4.9 \cdot 10^{-7}$ nm^2 corresponding to one MMC molecule was calculated as the circular projection of MMC molecular volume of $2.6 \cdot 10^{10}$ nm^3. With a laser spot size of 1.9 µm in diameter and taking the topography of the nanowires surface into consideration, the effective surface area probed by the laser was 1.7 μm^2. This gives an estimate of $N_{SERS} = 3.2 \cdot 10^6$ molecules. Hence, the calculated enhancement factor comes to $1.6 \cdot 10^6$.
